# Genetic Analyses of Rare ESBL ST628 *Klebsiella pneumoniae* Detected during a Protracted Nosocomial Outbreak in the United Kingdom

**DOI:** 10.3390/microorganisms12050883

**Published:** 2024-04-28

**Authors:** Stephen Mark Edward Fordham, Francis Drobniewski, Magdalena Barrow, Melissa Hutchings, Kate Crowther, Denise Richards, Paul Bolton, Anna Mantzouratou, Elizabeth Sheridan

**Affiliations:** 1 Department of Life & Environmental Sciences, Talbot Campus Fern Barrow, Bournemouth University, Poole BH12 5BB, UK; sfordham@bournemouth.ac.uk (S.M.E.F.); mbarrow@bournemouth.ac.uk (M.B.); amantzouratou@bournemouth.ac.uk (A.M.); 2 Department of Infectious Diseases, Hammersmith Campus, Imperial College London, 8th Floor, Office 8.N10, DuCane Road, London W12 ONN, UK; 3 Department of Medical Microbiology, Poole Hospital, University Hospitals Dorset NHS Foundation Trust, Longfleet Road, Poole BH15 2JB, UKpaul.bolton7@nhs.net (P.B.);

**Keywords:** *Klebsiella pneumoniae*, ESBL, plasmids, ST628, nosocomial, outbreak

## Abstract

*Klebsiella pneumoniae* (*K. pneumoniae*) cultures from a hospital-wide outbreak in the UK, which lasted for over 12 months, were sequenced. We sought to sequence and genetically characterise the outbreak strain. Antibiotic Susceptibility Testing (AST) was performed on 65 *K. pneumoniae* isolates saved from the outbreak. All isolates were sequenced using the Oxford Nanopore Technologies (ONT) MinION flowcell: 10 isolates, including the isolate with the earliest collection date in 2017, were additionally sequenced on the NovaSeq 6000 platform to build high-accuracy nanopore-illumina assemblies. Among the sequenced strains, 60 were typed as ST628. 96.6% (*n* = 58/60) ST628 strains harboured a large ~247-kb FIB(K) plasmid carrying up to 11 antimicrobial resistance genes, including the extended-spectrum beta-lactamase (ESBL) gene, *bla*_CTX-M-15_. Clonality between the outbreak isolates was confirmed using single nucleotide polymorphism (SNP) typing. The outbreak strains were phylogenetically related to clinical ST628 strains identified in 2012, 6 years prior to the outbreak. A rare ESBL *K. pneumoniae* K2 ST628 strain harbouring a multi-drug resistant (MDR) plasmid encoding the ESBL gene *bla*_CTX-M-15_ was detected across multiple independent wards during the protracted nosocomial outbreak. Surveillance of this strain is recommended to prevent future nosocomial outbreaks.

## 1. Introduction

Antimicrobial-resistant strains of *Klebsiella pneumoniae* (*K. pneumoniae*) encoding extended-spectrum β-lactamase (ESBL) genes are emerging globally and are associated with increased hospital stays, poor patient outcomes and rising healthcare costs [1]. Notably, third-generation cephalosporin-resistant *K. pneumoniae* (3GR-Kp) has been associated with increased in-hospital mortality and longer hospital stays [2]. Invasive 3GR-Kp has become widespread in the WHO European Region, with 18 countries reporting cephalosporin resistance rates ≥ 50% in invasive *K. pneumoniae* isolates [3]. Among a large-scale collection of invasive multi-drug resistant (MDR) *K. pneumoniae* isolates sourced from hospitals across the United Kingdom and Ireland, resistance to β-lactams was largely attributed to ESBLs. Notably, the ESBL encoding gene, *bla*_CTX-M-15,_ was gained by phylogenetically distinct MDR *K. pneumoniae* lineages. This gene, alongside *bla*_SHV_ variants and the rare *bla*_CTX-M-26_ gene, accounted for 95% of the ESBL phenotype and was strongly associated with resistance to β-lactams antibiotics [4].

Among the ESBLs in *K. pneumoniae*, a high prevalence of cefotaximases, CTX-M serine-β-lactamases have been recorded: notably, the CTX-M-15 enzyme, a product of the *bla*_CTX-M-15_ gene has been reported worldwide [5,6,7]. The *bla*_CTX-M-15_ gene is often found to be associated with the insertion sequence (IS), IS*Ecp1* [8]. IS*Ecp1* may have captured the presumed ancestor of *bla*_CTX-M-15_, *bla*_CTX-M-3_ from the chromosome of *Kluyvera* spp. [9]. IS*Ecp1* can mobilise and lead to the expression of CTX-M-type ESBLs, including *bla*_CTX-M-15_, between plasmids and chromosomes within the Enterobacteriaceae family.

Typically, *bla*_CTX-M-15_ is encoded on incompatibility type F conjugative plasmids in Enterobacterales, particularly in *K. pneumoniae* [6,8,10]. During a year-long surveillance survey, an IncF conserved plasmid encoding *bla*_CTX-M-15_, called Plasmid A, was identified in 4 *K. pneumoniae* STs (ST323, ST29, ST5822, and ST221), and *K. varicola* ST347 [7]. Furthermore, despite the reinforcement of hygiene and infection control protocols in a French intensive care unit (ICU) during the COVID-19 pandemic, an outbreak of *K. pneumoniae* ST394-producing CTX-M-15 occurred [11]. The *bla*_CTX-M-15_ gene is an important determinant for the epidemic success of outbreak *K. pneumoniae* isolates, representing a significant challenge in healthcare settings.

A clonal outbreak in the UK of an otherwise uncommon ST recorded worldwide, ST1788 encoding *bla*_CTX-M-15_, has recently been reported [12]. The ST1788 *bla*_CTX-M-15_ containing strain was detected across 11 hospitals and 11 general practices in Southern and Western Wales, UK, between February 2019 and November 2021 [12]. In 2018, another nosocomial outbreak of an ESBL *K. pneumoniae* strain was detected from an acute hospital in Dorset, UK (See outbreak description). Whole genome sequencing (WGS) of isolates saved from this period was used to assemble and characterise the outbreak strain and confirm the clonal spread of the strain across independent hospital departments to better inform the epidemic potential of CTX-M-15 encoding strains in the UK.

The presence of *bla*_CTX-M-15_ on a large type F plasmid in *K. pneumoniae* clones from a 2018 outbreak at an acute hospital in Dorset in the UK is reported. The *K. pneumoniae* clone, ST628, has not previously been associated with a CTX-M-outbreak. We present WGS data from 65 sequenced strains saved from the outbreak period, 2017 to 2018, including a sample from 2017, the earliest date of ESBL *K. pneumoniae* detection, and 60 and 4 samples with an ESBL phenotype from 2018 and 2022, respectively.

## 2. Outbreak Description

In early March 2018, an intensive care nurse (ICN) identified 2 patients in the same trauma ward with a new diagnosis of ESBL *K. pneumoniae,* which were both resistant to gentamicin. Given the unusual antibiogram and the fact that the patients were connected by time and place, the consultant microbiologist sent the isolates to Public Health England (PHE) for typing. Typing confirmed a unique VNTR profile that had not been previously recorded in the UK. The consultant microbiologist requested additional screening for other patients who had been in contact with the cases. This yielded further cases, and the decision was made to screen the entire trauma ward. Further screening of patients across hospital wards confirmed that the strain was not limited to one ward.

The identification of the same strain beyond the trauma ward led to the initiation of a formal screening program on 6 May 2018. Confirmed cases included a positive isolate of ESBL *K. pneumoniae* recorded by the Hospital Microbiology services team with a specific VNTR profile with a specimen date after 1 October 2017. Probable cases were similarly defined as a positive isolate of ESBL *K. pneumoniae* that were also resistant to gentamicin, ciprofloxacin, and either resistant or intermediately resistant to trimethoprim and tazocin, respectively. The screening was stopped when no new positive cases were identified for three consecutive months, and good infection control practices were applied. During the course of the outbreak, 28,683 screening samples were taken from 12,752 patients. From the screening and clinical samples collected during the outbreak, 262 patients with the outbreak strain were identified. Of the 262 patients identified with the outbreak strain, 10 were identified from blood samples, 59 from urine samples, and 166 cases were identified from rectal/stool samples.

Sixty-one isolates were saved from the outbreak from July 2017 to December 2018, and four isolates from 2022 with the same antibiogram were sequenced in order to genetically characterise the strains and identify if related strains existed prior to the outbreak. No *K. pneumoniae* strains from the period 2018 to 2022 had an antibiogram similar to those collected in 2018. As such, from the period 2018 to 2022, no *K. pneumoniae* isolates were saved for sequencing.

## 3. Materials and Methods

### 3.1. Culture

Stored isolates saved from the outbreak were thawed on a blood agar plate (Becton-Dickinson [BD], Sparks, NV, USA). This was performed by removing one of the pearls from the small vial with glass cryopearls stored at −80 °C in 15% glycerol using a sterile loop and placing it on the surface of an agar plate. The pearl was then rolled around the surface of the plate. The plate was incubated at 37 °C for 24 h.

A single distinct colony from each strain was added onto a Matrix-Assisted Laser Desorption/Ionization (MALDI) target plate. A total of 1 µL of α-Cyano-4-hydroxycinnamic acid (CHCA) matrix solution prepared with 50% acetonitrile and 2.5% trifluoroacetic acid in pure water was spotted onto the plate. A Matrix-Assisted Laser Desorption/Ionization Time-of-Flight Mass Spectrometry (MALDI-TOF MS) (Bruker Daltonics, Billerica, MA, USA) assignment score ≥ 2.0 was used to confirm species identification.

For culture, a single pure colony was inoculated into 10 mL of lysogeny broth (LB) containing 8 µg/mL cefotaxime (Sigma-Aldrich, Southampton, UK) in a 50 mL centrifuge tube to permit aerobic respiration. Culturing was performed for 24 h at 37 °C before downstream DNA extraction. The same culture was used to prepare a bacterial lawn for antibiotic susceptibility testing (AST).

To determine both the ceftriaxone and cefotaxime minimum inhibitory concentration (MIC), 2–3 morphologically similar bacterial colonies were lightly touched using the Prompt inoculation system wand ( (bioMérieux SA, Marcy l’Etoile, France) and then suspended thoroughly into a blank saline solution. The turbidity of the inoculum in the saline solution was standardised to 0.5 McFarland units. A sterile swab was then inserted into the bacterial suspension before being streaked onto a Mueller-Hinton agar plate to create a bacterial lawn. The BioMérieux ETEST AST reagent strips, ceftriaxone (ceftriaxone TXL 0.002–32 µg/mL), and cefotaxime (cefotaxime CTL 0.002–32 µg/mL) were added to the plate aseptically. The plates were incubated at 37 °C for 24 h. The MIC value was recorded where the pointed end of the eclipse intercepts the strip.

### 3.2. Genomic DNA Extraction, Library Preparation, and Sequencing

Bacterial genomic DNA was extracted using the Wizard^®^ HMW DNA extraction kit from Promega (Southampton, UK) as described by the manufacturer. Long-read library preparation for all 65 strains was performed using the PCR-free Rapid sequencing gDNA barcoding kit, SQK-RBK004, following the protocol provided by Oxford Nanopore Technologies (ONT) (Oxford, UK). The prepared library was loaded onto a R9.4.1 flowcell (ONT). Raw Fast5 files were basecalled using Guppy version 6.3.8, using the high-accuracy, r9.4.1_450bps_hac model, with the adapter trimming option. The reference type strain is *Klebsiella pneumoniae* subsp. *pneumoniae* (ATCC 13883), with a known genomic sequence, was barcoded for each sequencing run to ensure reads deposited into barcode bins could faithfully assemble known contigs to closure with a high consensus accuracy > 99%.

For high typing resolution, 10 genomes were assembled using both nanopore long reads and illumina short reads. Ten representative strains (one strain collected in 2017, the earliest year of the outbreak, eight samples from 2018, and a further sample detected in 2022) were sequenced on the NovaSeq 6000 platform (Illumina, San Diego, CA, USA), generating 150-bp paired-end (PE) reads. These strains included: UHD-56 (2017), UHD-33, UHD-41, UHD-45, UHD-49, UHD-50, UHD-53, UHD-57, UHD-58 (2018), and UHD-4 (2022), respectively (Supplementary File S1).

Illumina libraries were prepared using the xGen™ DNA EZ Library Prep Kit (Agilent Technologies, Palo Alto, CA, USA) (according to the manufacturer’s protocol. Libraries were indexed using xGen UDI 10nt Primers (Agilent Technologies, Palo Alto, CA, USA). The prepared libraries were quantified via a fluorometric method using the Invitrogen Qubit dsDNA assay and qualified using electrophoretic separation on the Agilent TapeStation 4200 (Agilent Technologies, Palo Alto, CA, USA).

Fastp (https://github.com/OpenGene/fastp, accessed on 18 September 2023) was used to trim reads, remove adapter sequences, and perform quality filtering on the raw reads. A default Phred quality score of 15 over a sliding window size of 4 bp was applied to the raw reads. Filtered reads were subsequently used for genome polishing. Nanopore-illumina assemblies were used to confirm clonality between the isolates. An SNP distance ≤ 20 was used to confirm clonality between the outbreak *K. pneumoniae* isolates.

*De novo* genome assembly was performed using Flye v2.9 [13], following light read filtering, which discarded reads < 1000 bp. Graphical Fragment Assembly (GFA) files for each final genome assembly were visualised using Bandage v0.8.1 to confirm circularity [14]. Draft genome assemblies underwent two consecutive rounds of Medaka v1.6.0 post-assembly polishing (https://github.com/nanoporetech/medaka, accessed on 18 September 2023).

For Nanopore-illumina assemblies, medaka polished long-read assemblies were subsequently polished using shorts reads using Polypolish v.0.5.0 [15]. *K. pneumoniae* chromosomes are given a Universal hospital designation number (UHD-*n*). Their matching plasmid is designated, pESBL-PH-number (pESBL-PH-*n*).

### 3.3. Antibiotic Susceptibility Testing (AST)

Antibiotic Susceptibility Testing (AST) on all sequenced ESBL strains was performed using the Kirby-Bauer disc diffusion assay. Interpretation of sensitivity zone diameter breakpoints was based on the 2024 European Committee on Antimicrobial Susceptibility Testing (EUCAST) Version 14.0 document guidelines (https://www.eucast.org/clinical_breakpoints, accessed on 4 January 2024). Multidrug resistance (MDR) *K. pneumoniae* strains were defined by either non-susceptibility/resistance to at least one agent from three or more antibiotic classes [16]. Both *E. coli* ATCC 25922 and *Klebsiella pneumoniae* ATCC 13883 were used as antimicrobial-sensitive negative control strains, while *Klebsiella pneumoniae* K6 ATCC 700603 served as a β-lactam-positive control strain.

AST testing was performed for each strain using the following 11 Oxoid (Southampton, UK) antimicrobial susceptibility discs: amoxicillin-clavulanic (AMC): 30 µg, ampicillin (AMP): 10 µg, ceftazidime (CAZ): 10 μg, ciprofloxacin (CIP): 5 µg, gentamicin (CN): 10 μg, cefpodoxime (CPD): 10 μg, cefuroxime (CXM): 30 μg, meropenem (MEM): 10 μg, piperacillin-tazobactam (TZP): 36 μg, trimethoprim-sulfamethoxazole (SXT): 25 μg, and tobramycin (TOB): 10 µg.

The MIC for both the third-generation cephalosporins, ceftriaxone (ceftriaxone TXL 0.002–32 µg/mL), and cefotaxime (cefotaxime CTL 0.002–32 µg/mL) was determined for 12 strains using the BioMérieux ETEST reagent strips. The 12 strains included all illumina-nanopore assemblies: UHD-56 (2017), UHD-33, UHD-41, UHD-45, UHD-49, UHD-50, UHD-53, UHD-57, UHD-58 (2018), and UHD-4 (2022), respectively. In addition, the MIC for both ceftriaxone and cefotaxime was determined for strains UHD-64 and UHD-65. These two strains were selected for cephalosporin MIC determination because nanopore sequencing did not identify a plasmid from these assemblies, and they were typed to ST628 with the same VNTR profile as the outbreak strains (Supplementary File S1). Interpretation of MIC breakpoints was determined using the 2024 EUCAST Version 14.0 breakpoint tables for Enterobacterales. Both *E. coli* ATCC 25922 and *Klebsiella pneumoniae* ATCC 13883 were used as ESBL-negative control strains, while *Klebsiella pneumoniae* K6 ATCC 700603 served as an ESBL-positive control strain.

### 3.4. Klebsiella Pneumoniae Typing

For the 10 nanopore-illumina assemblies, Kleborate v.2.3.2 was used for K (capsule) and O antigen (LPS) serotype prediction [17]. In addition, multi-locus sequence type (MLST) designation for all isolates was assigned using Kleborate v.2.3.2 [17]. In-silico antimicrobial resistance prediction was performed using ResFinder 4.0 with a minimum nucleotide identity and coverage of 99% [18]. MobileElementFinder v.1.0.2, available from the Center for Genomic Epidemiology (https://www.genomicepidemiology.org/, accessed on 25 September 2023), was used to identify mobile genetic elements (MGEs) and their relationship with AMR genes. Putative virulence and persistence-associated genes were identified via bacterial whole genome annotation using Prokka v1.14.6 [19]. The BlastP program from NCBI was used to determine the identity and coverage of these gene products, employing a minimum threshold of 99% for both of these parameters. Finally, plasmid replicon typing was performed using PlasmidFinder, employing a minimum identity and coverage of 98% [20]. Single nucleotide polymorphisms (SNPs) distance was determined using the rapid haploid variant calling program Snippy (https://github.com/tseemann/snippy, accessed on 25 September 2023).

### 3.5. Phylogenetic Tree Construction

The phylogenetic tree was constructed using the Phylogenomics bacterial genome tree tool available from the Bacterial and Viral Bioinformatics Resource Center (BV-BRC) (https://www.bv-brc.org/, accessed on 11 November 2023). For tree construction, 10 UK outbreak strains, eight phylogenetically related strains identified using BacWGSTdb core genome multi-locus sequence typing (cgMLST) tool, and 5 ST628 isolates recovered from a patient with relapsed acute myeloid leukaemia (AML) admitted to Kyushu University Hospital, Japan was used [21]. To build the tree, 1000 non-duplicate genes common to each genome were chosen to build a concatenated alignment. The Randomized Axelerated Maximum Likelihood (RAxML) program was used to build the tree [22]. Tree support values were generated using 100 rounds of rapid bootstrapping.

### 3.6. Data Availability Statement

The 10 nanopore-illumina genome assemblies can be found on NCBI under the BIOPROJECT number PRJNA949681, available: https://www.ncbi.nlm.nih.gov/search/all/?term=PRJNA949681, accessed on 11 November 2023.

For genome meta-data, consult Supplementary File S1.

## 4. Results

**Figure 1:**
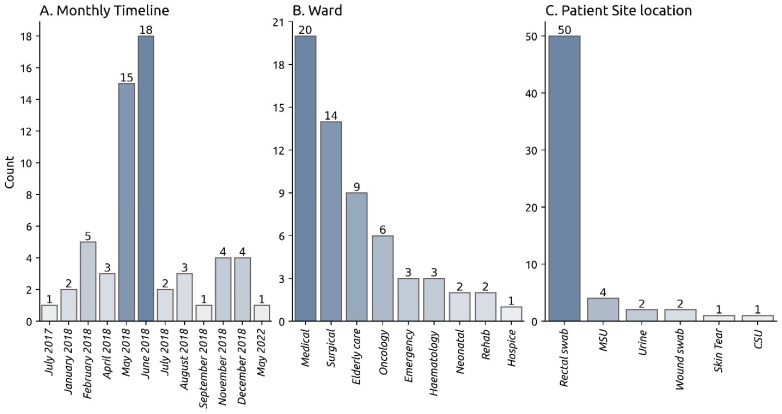
Monthly timeline, ward, and site location of the sequenced ST628 *K. pneumoniae* strains.

Sixty-five strains saved from the outbreak period underwent sequencing. 92.3% (*n* = 60/65) of these strains were typed either to ST628 or a ST628 locus variant via nanopore-only sequencing. 98.3% (*n* = 59/60) of the ST628 strains had a recorded collection date (Supplementary File S1). The strains collection dates ranged from 15 July 2017 (UHD-56) to 31 May 2022 (UHD-4), while the remaining isolates were collected from individual patients from January 2018 through to December 2018. The majority, 55.0% (*n* = 33/60), were collected between May and June 2018 (Figure 1A). The ST628 strains were sourced from 9 unique hospital departments (Figure 1B). Notably, 33.33% (*n* = 20/60) and 23.33% (*n* = 14/60) were identified as patients from either the medical or surgical wards, respectively. Notably, 5% (*n* = 3/60) of the ST628 strains were detected in patients from the Emergency department. The majority, 83.33% (*n* = 50/60) of the sequenced ST628 strains were derived from rectal swab samples (Figure 1C) obtained from ESBL *Klebsiella pneumoniae* screening. Seven clinical samples were obtained from urine specimens, while two samples were sourced from an infected C-section and an open skin tear, respectively.

### 4.1. Resistance Rates of Isolates Saved from the Outbreak

Resistance against 11 antimicrobials was measured using the Kirby-Bauer disc diffusion assay. All *Klebsiella* hospital strains 100% (*n* = 65/65) were resistant to ampicillin, and susceptible to the carbapenem, meropenem with a resistance rate of 95.3% (*n* = 62/65) to cefuroxime, cefpodoxime, and amoxicillin-clavulanic acid, 93.84% (*n* = 62/65) towards ceftazidime, sulfamethoxazole-trimethoprim, and piperacillin-tazobactam, 92.3% (*n* = 60/65) for ciprofloxacin and gentamicin and 90.7% (*n* = 59/65) for tobramycin, respectively. Genome meta-data and resistance categorisation based on AST can be found in Supplementary File S1.

The BioMérieux ETEST MIC confirmed all 10 nanopore-illumina assemblies had a resistant phenotype to both ceftriaxone and cefotaxime, surpassing the 2 mg/L thresholds for Enterobacterales. The MIC was >32 mg/L for both ceftriaxone and cefotaxime, confirming a resistant phenotype towards third-generation cephalosporins (3GRs). Interestingly, the MIC for both UHD-64 and UHD-65 was <1 mg/L, yielding a sensitive phenotype.

### 4.2. AMR Genotype of Isolates Saved from the Outbreak

Sixty strains were typed as *K. pneumoniae* ST628 or a locus variant of ST628 via nanopore-only long-read sequencing; 96.6% (*n* = 58/60) harboured an MDR plasmid. These strains harboured either 11 (*n* = 51/58), 10 (*n* = 1/58), 9 (*n* = 3/58), 8 (*n* = 1/58), 7 (*n* = 1/58) or 5 (*n* = 1/58) AMR genes, respectively on a plasmid (Supplementary File S1). The complement of 11 resistance genes was encoded on a single large FIB(K) plasmid with a median size of 246,833-bp. The AMR genes in genetic co-linkage included: *aac(3)-IIe*, *aac(6*′*)-Ib-cr*, *strA*, *strB*, *bla*_CTX-M-15_, *bla*_OXA-1_, *bla*_TEM-1B_, *dfrA14*, *qnrB1*, *sul2*, and *tet(A)*, respectively. Additionally, 2 ST628 strains recorded during the outbreak period, UHD-64 and UHD-65, sensitive to all antimicrobials with the exception of ampicillin, revealed these strains lacked a resistance encoding plasmid. Resistance to 6 drug classes: diaminopyrimidine, sulfonamides, fluoroquinolones, cephalosporins, tetracyclines, and aminoglycosides, respectively, is thereby likely conferred by the outbreak plasmid.

The 11 AMR genes encoded resistance to 6 drug classes. These drug classes and the corresponding gene conferring resistance included diaminopyrimidine (*dfrA14*), sulfonamides (*sul2*), fluoroquinolone (*qnrB1*, *aac(6*′*)-Ib-cr*), cephalosporins (*bla*_CTX-M-15_, *bla*_OXA-1_, *bla*_TEM-1B_), aminoglycosides (*aac(3)-IIe*, *aac(6*′*)-Ib-cr*, *strA*, *strB*), and tetracyclines (*tet(A)*), respectively. A ~15.3-kb pseudo-compound transposon (PCT) bracketed by IS*26* insertion sequences in the same orientation, encoding 5 AMR genes, was typed in the ST628 strains, with the exception of pESBL-PH-7 which harbours the IS26 PCT but lacks *bla*_CTX-M-15_. Notably, pESBL-PH-7 was susceptible to cefuroxime, cefpodoxime, and ceftazidime, respectively. This may suggest the absence of the *bla*_CTX-M-15_ gene confers a susceptible phenotype towards second- and third-generation cephalosporins. The IS*26* PCT detected in the other strains encoded *bla*_TEM-1B_, *sul2*, *strA*, *strB*, and the ESBL *bla*_CTX-M-15_, respectively. 100% nucleotide identity and coverage for IS*26* (accession: X00011), including the 14-bp terminal inverted repeats (TIRs), were identified within the IS*26* PCTs (Supplementary Figure S1).

### 4.3. Nanopore-Illumina Assemblies of 10 Representative ST628 Outbreak Isolates

**Figure 2:**
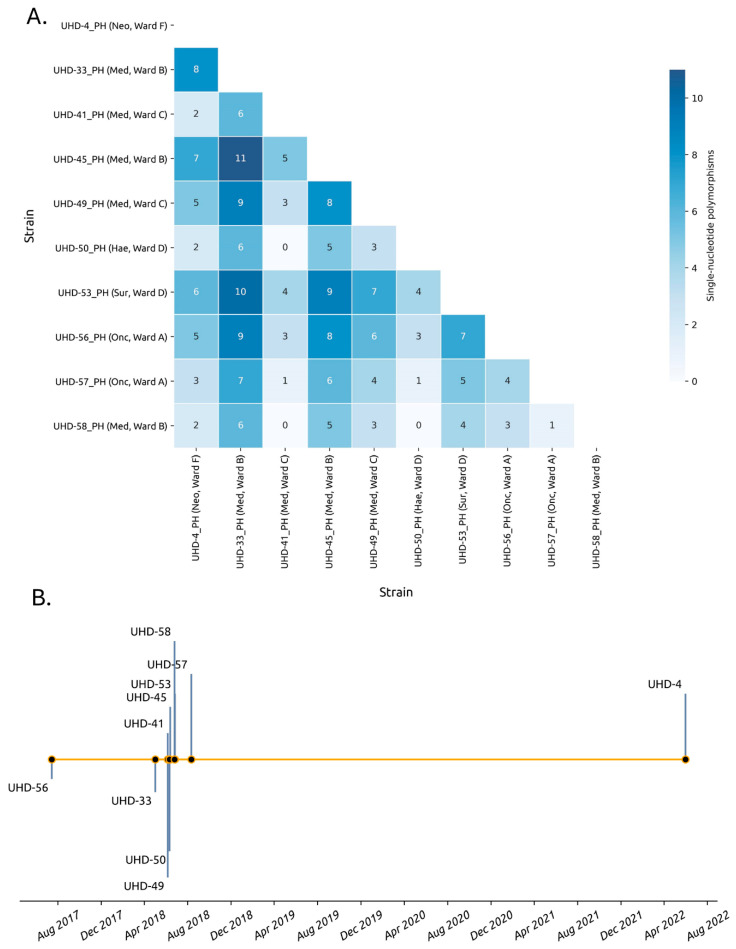
All-versus-all SNP comparison between the *K. pneumoniae* strains sequenced via both nanopore and illumina platforms. (**A**) Hospital departments are abbreviated by a three-letter code. Neo: neonatal, med: medical, hae: haematology, sur: surgical, onc: oncology. (**B**) Timeline collection.

Ten strains from the outbreak underwent sequencing via both nanopore and illumina platforms to determine the genetic relatedness of the isolates between separate hospital wards and the relatedness of the strains to the UHD-56 strain collected in 2017. All of the strains were typed as ST628 with a K2 locus (KL2) and a O3b locus (O3b). The SNPs between these strains were compared against one another (Figure 2A). Notably, all of the strains were closely related to one another, with an SNP difference ≤ 11, despite a collection date ranging from 15 July 2017 (UHD-56_PH) to 31 May 2022 (UHD-4_PH). In addition, all strains were ≤9 SNPs distant from UHD-56_PH, the isolate with the earliest known collection date. Among the 10 nanopore-illumina strains sequenced, the isolates were sourced from 6 unique ward types belonging to 5 ward departments: neonatal, medical, haematology, surgical and oncology, respectively. Interestingly, no SNPs were detected between UHD-41_PH (medical, ward C), UHD-50_PH (haematology, ward D), and UHD-58_PH (medical, ward B), despite their identification across three independent wards separated by space (Figure 2A). These three strains were, however, collected within 19 days of one another (Figure 2B), suggesting the spread of a single clone at a point in time across multiple departmental wards. The narrow SNP distance between all isolates also suggests a single clone was responsible for the outbreak across independent departments. Assemblies can be found on NCBI under the BIOPROJECT number PRJNA949681. Candidate virulence and persistence genes and their functions encoded by the *K. pneumoniae* chromosomes are shown in Table 1.

### 4.4. Phylogenetically Similar ST628 K. pneumoniae Strains

**Figure 3:**
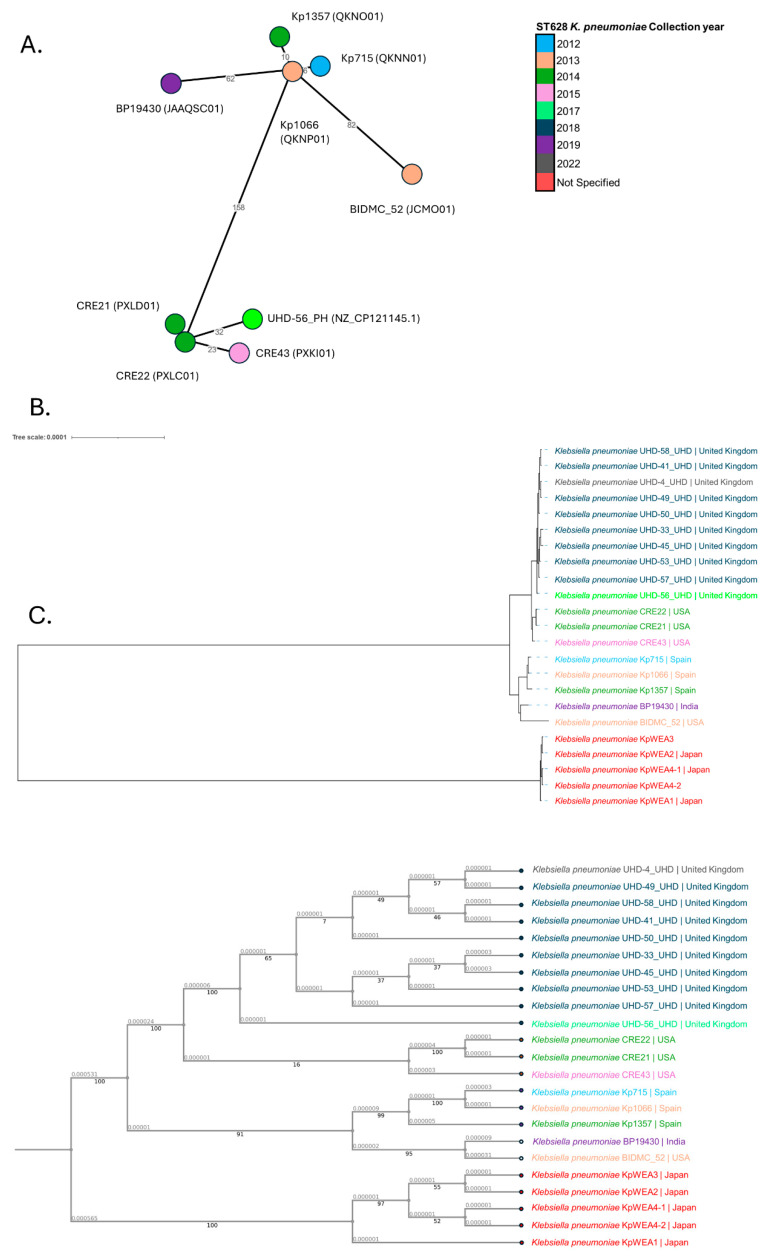
Phylogenetic analysis of ST628 *K. pneumoniae* strains. (**A**) cgMLST identified eight phylogenetically related strains against the 2017 UHD-56_PH index strain. All strains, with the exception of BIDMC_52, belong to ST628. (**B**) CRE22, -21 and -43 from Palo Alto, California, USA, were the closest related strain to UHD-56_PH. The KpWEA*n* isolates form a distinct cluster to the outbreak and other ST628 strains. The unit of branch length represents nucleotide substitutions per site. (**C**) The cladogram was constructed using the phylogenetically related 8 ST628 strains, the UHD-*n*_PH outbreak strains and the KpWEA*n* strains. Distinct clades are formed, mirroring the relationship depicted in the phylogenetic tree. Branch length is indicated as a decimal number. Bootstrap support values are displayed as integers, generated using 100 rounds of the “Rapid” bootstrapping option [22]. The colours in the key refer to the year of isolation.

Phylogenetically related ST628 *K. pneumoniae* strains dating from 2012, 6 years prior to the hospital outbreak, have been discovered. The *K. pneumoniae* strain, UHD-56_PH from 2017, was set as a query sequence on the BacWGSTdb server to identify closely related strains. BacWGSTdb employs a cgMLST scheme utilising 2358 cgMLST loci. Here, eight strains from three unique countries were identified. These included four strains from the USA, three from Spain and one from India. Notably, all of these strains were ST628, with the exception of BIDMC_52, identified in 2013 from Boston, MA, USA, which belonged to ST3615. According to the cgMLST scheme, three clustered clinical isolates from the USA were the closest related strains to UHD-56_PH. These strains included CRE22 (accession: PXLC01), CRE21 (accession: PXLD01), and CRE43 (accession: PXKI01), collected from Palo Alto, California, USA in November 2014 (CRE21/22) and December 2015 (CRE43). Relative to UHD-56_PH, CRE22 differed by only 32 cgMLST loci (Figure 3A) despite a 3-year earlier collection date in a geographically distinct area.

The SNP distance between UHD-56_PH and the related isolates identified using cgMLST was further investigated. In agreement with the cgMLST results, the three CRE isolates from California were the closest related strains. CRE21, CRE22, and CRE43 were 91, 94 and 289 SNPs distant from UHD-56_PH. This relationship is further confirmed in both the phylogenetic tree and cladogram whereby the CRE21/22/43 isolates cluster together closest to the UHD-*n*_PH strains collected from our UK district general hospital from 2017 to 2022 (Figure 3B,C). The three clustered clinical strains from Spain were also related to UHD-56_PH; Kp715 (NCBI RefSeq assembly: GCA_003225855.1), Kp1066 (NCBI RefSeq assembly: GCA_003226115.1), and Kp1357 (NCBI RefSeq assembly: GCA_003226075.1) had a SNP distance of 558, 565, and 578, respectively. The remaining ST628 isolates, each forming distinct clusters, were distal to UHD-56_PH. BP19430 (NCBI RefSeq assembly: GCA_011742315.1) from India in 2019, and the KpWEA*n* isolates collected from Japan were 1793, and between 8713 (KpWEA2, accession: NZ_AP024571.1), and 8751 SNPs (KpWEA4-1, accession: NZ_AP024577.1) SNPs distant. Interestingly, for all ST628 strains, with the exception of both BP19430 and the KpWEA*n* strains, the virulence-associated K2 capsule was present.

### 4.5. Plasmid in Outbreak ST628 K. pneumoniae Strains

**Figure 4:**
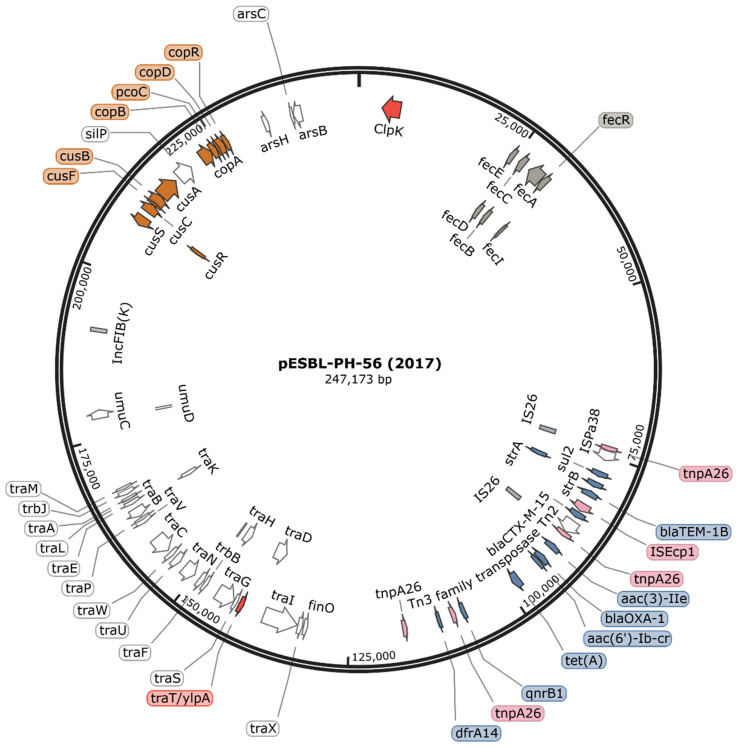
Prototypical reference plasmid, pESBL-PH-56. pESBL-PH-56 was identified in 2017 from a rectal swab sample from Ward A, from our acute hospital in Dorset, UK. pESBL-PH-56 was isolated from a 5,316,664-bp ST628 K2, O3b *K. pneumoniae* strain. pESBL-PH-56 is a FIB(K) 247,173-bp plasmid encoding *tra* conjugative transfer genes (white), copper resistance genes (copper), arsenic resistance genes (white), the ferric citrate iron acquisition system (grey), virulence genes: *traT*/*ylpA* and *clpK* (red), and antimicrobial resistance genes (blue). Flanking IS*26* insertion sequences are shown in grey, and their corresponding transposase gene, *tnpA26*, is shown in pink.

Two virulence genes were located in all high-resolution nanopores and illumina pESBL-PH-*n* plasmids. The thermo-tolerant Clp ATPase, *clpK,* was present on the outbreak plasmid. ClpK has been implicated in conferring a thermoresistant phenotype, enabling bacteria to survive common disinfection protocols encountered in the hospital environment and enabling nosocomial persistence. Furthermore, the virulence factor TraT, a transfer protein that inhibits the classical pathway of complement activation, was identified. An overview of the resistance, virulence, and persistence genes is shown in plasmid pESBL-PH-56 in Figure 4 and Table 2.

The 10 nanopore-illumina assemblies possessed the incompatibility replicon FIB(K). All the assembled plasmids were highly similar. No SNPs were identified between all plasmid samples and pESBL-PH-56. Taken together, these results confirm the outbreak was caused by a FIB(K) AMR plasmid-carrying bacterium.

All plasmids, with the exception of pESBL-PH-4, were within ≤14-bp from the length of the 247,173-bp pESBL-PH-56 plasmid. Plasmid pESBL-PH-4 had a length of 192,395-bp, 54,778-bp shorter than pESBL-PH-56. pESBL-PH-4 was identified from a midstream urine specimen (MSU) from a patient with an infected C-section. This sample was collected in 2022, 4 years after the 2017/2018 outbreak.

### 4.6. Similar Plasmids from the ST628 K. pneumoniae Strains

**Figure 5:**
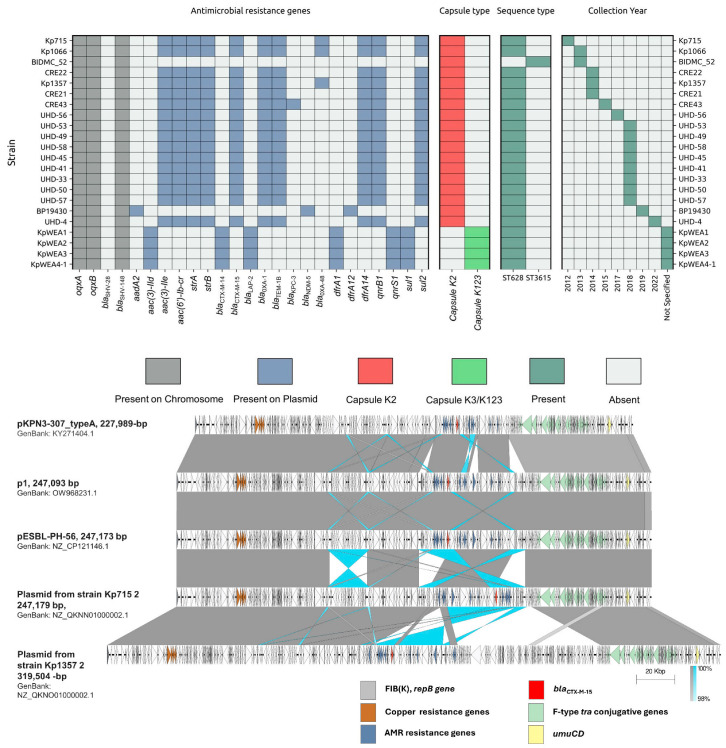
Analysis of plasmids from ST628 *K. pneumoniae* strains. (**Top**) Meta-data pertaining to each of the ST628 strains. For each of the strains, the AMR gene complement, capsule type, ST, and year of collection are shown. Kp715 and Kp1357 harbour the same plasmid-encoded resistance genes as those present in the UHD-*n*_PH samples. All of the ST628 strains additionally encode the virulent K2 capsule, with the exception of BIDMC_52, which belongs to a separate ST and the phylogenetically distinct KpWEA*n* clustered isolates, which belong to capsule type K123. (**Bottom**) An almost identical plasmid backbone between pESBL-PH-56 and the Kp715 FIB(K) 247,179-bp plasmid was collected in 2012 from Barcelona, Spain. This backbone is also similar to plasmids from *K. pneumoniae* ST307 isolates, p1, and pKPN-307_typeA. Common gene features are depicted in the key. A plasmid comparison map was produced using EasyFig v.2.2.2 [23].

pESBL-PH-56 from the *K. pneumoniae* strain UHD-56_PH was compared to both plasmids isolated from phylogenetically similar ST628 strains and plasmids isolated from other *K. pneumoniae* strains. Interestingly, the CRE*n* strains from California possessed the same 11 AMR genes encoded on pESBL-PH-56. These included: *aac(3)-IIe*, *aac(6*′*)-Ib-cr*, *strA*, *strB*, *bla*_CTX-M-15_, *bla*_OXA-1_, *bla*_TEM-1B_, *dfrA14*, *qnrB1*, *sul2*, and *tet(A)*, respectively (Figure 5). The FIB(K) replicon was also identified from the CRE*n* assemblies. WGS on the CRE strains was performed using only illumina short-reads, which precluded the assembly of complete circular plasmid contigs, producing assemblies with contigs ranging from 293 for CRE22, 304 for CRE21, and 1060 for CRE43, respectively. Despite this, the phylogenetic similarity, the same complement of AMR genes, and the detection of the same plasmid FIB(K) replicon implies the CRE*n* strains were both closely related and possessed a similar plasmid to the outbreak UHD strains.

Plasmids derived from two other phylogenetically related ST628 *K. pneumoniae* strains were compared against pESBL-PH-56. Interestingly, strain Kp715 harboured a FIB(K) 247,179-bp plasmid encoding the same 11 AMR genes as those present on pESBL-PH-56. This strain was collected in 2012 from Barcelona, Spain, 5 years prior to the isolation of UHD-56_PH harbouring pESBL-PH-56. Both plasmids have an almost identical plasmid backbone and length (Figure 5). The 247,179-bp plasmid (accession: NZ_QKNN01000002.1) has 100% coverage and 99.98% nucleotide identity against pESBL-PH-56. In addition to this plasmid, ST628 strain Kp1357 harboured a FIB(K) 319,504-bp plasmid encoding the same set of 11 AMR genes as both pESBL-PH-56 and the plasmid from Kp715 (Figure 5). Kp1357 was also similar to pESBL-PH-56; 91% coverage and 99.99% nucleotide identity. Similar plasmid backbones are shown in Figure 5. Interestingly, both the Kp715 and Kp1357 strains additionally encoded the carbapenemase *bla*_OXA-48_ on a separate IncL 63,589-bp plasmid, despite being collected 21 months apart; Kp715 (August 2012), Kp1357 (May 2014) suggesting ST628 strains may be receptive towards uptake and maintenance of an IncL plasmid encoding a carbapenemase gene.

## 5. Discussion

This is the first reported clonal outbreak of K2 ST628 *K. pneumoniae*. The ST628 strains harboured an ~247-kb FIB(K) plasmid encoding 11 AMR genes: *aac(3)-IIe*, *aac(6*′*)-Ib-cr*, *strA*, *strB*, *bla*_CTX-M-15_, *bla*_OXA-1_, *bla*_TEM-1B_, *dfrA14*, *qnrB1*, *sul2*, and *tet(A)*. Despite this, phylogenetically related clinical ST628 strains have been discovered from distinct geographic areas and temporal periods. CRE22, -21, and -43 strains were collected from clinical samples by the Stanford Healthcare Clinical Microbiology Laboratory in Palo Alto, California, USA in 2014/15, while Kp715 (2012), Kp1066 (2013), and Kp1357 (2014) strains have been collected from the Hospital Universitari Vall d’Hebron. Each of these strains also encoded the virulence-associated K2 capsule, identified from the outbreak isolates collected from our UK district general hospital.

Among the phylogenetically related strains, an almost identical FIB(K) plasmid encoding the same 11 AMR genes present on pESBL-PH-*n* was identified. For the ST628 K2 CRE*n* isolates, sequencing was performed using illumina short reads, which prevented the assembly of complete plasmids. Despite this, the same complement of 11 AMR genes and the same FIB(K) replicon family was identified, inferring that the CRE*n* strains were not only phylogenetically related to the UK district general hospital outbreak strains but also harboured a similar MDR plasmid. In addition, both Kp715 and Kp1357 harbour complete FIB(K) plasmids encoding the same 11 AMR genes as those encoded by pESBL-PH-*n*. The fact that both phylogenetically close CRE-21, -22, -43, and Kp715, -1357 ST628 isolates were discovered before the outbreak and encoded virtually identical plasmids suggests the UK outbreak isolates may be derived from these strains. Both ST628 strains BP19430 collected from India in 2019 and the KpWEA*n* isolates collected from Japan encoded distinct AMR genes from the outbreak ST628 *K. pneumoniae* strains [21], suggesting they were unrelated. Taken together, these results suggest the closest strain to the outbreak UHD-PH strain belongs to the 2014–15 CRE21/22/43 cluster collected from Palo Alto, California, USA, but were also similar to Kp715 and Kp1357 strains harbouring similar plasmids identified before the outbreak in 2012 and 2014.

The identification of K2 ST628 Kp715 encoding an almost identically sized plasmid may suggest remarkable plasmid conservation over a period of time spanning 5 years. Notably, a *bla*_CTX-M-15_ encoding IncF plasmid, named plasmid A, with a backbone similar to pKPN-307, has persisted in 3 plasmid A positive strains for 3–6 years in a hospital setting, confirming the long-term maintenance of a single CTX-M-15 encoding plasmid [7].

A narrow SNP distance ≤ 11 was detected among the 10 *K. pneumoniae* ST628 strains sequenced using both nanopore and illumina platforms. Furthermore, no SNPs were detected among three isolates sourced from 3 independent hospital wards. Collectively, these results suggest a single clonal strain was responsible for the outbreak. Similar strains detected in separate wards may imply the probable cause of transmission was the hands of healthcare workers, inadequate cleaning of equipment or the environment, and direct transmission through the use of shared bathrooms. Hands of healthcare workers have recently been implicated in the nosocomial spread of carbapenem-resistant (CR) ST11 K57 *K. pneumoniae* in China [24], while transmission of indistinguishable ESBL-producing ST307 *K. pneumoniae* strains encoding CTX-M-15 from sinks and drains in a surgical ward at Tokyo Medical University Hospital has recently been reported [25].

Clonal outbreaks of strains harbouring *bla*_CTX-M-15_ encoding IncF plasmids have been reported in other *K. pneumoniae* STs, such as ST307, ST1427, ST29, ST323, and *Klebsiella varicola* ST347 [5,7,26,27]. ST628 strains recovered from the outbreak hospital may represent another receptive *K. pneumoniae* strain with epidemic potential capable of maintaining a large IncF conjugative MDR plasmid. WGS confirmed the clonality between the strains. Nosocomial-linked transmission is strongly associated with the carriage of ESBL genes [28]. The plasmid-encoded *bla*_CTX-M-15_ may have played a role in the hospital outbreak recorded.

The plasmid with the earliest isolation date (2017), pESBL-PH-56, shares homology with previously reported plasmids, including pKPN3-307_typeA, found on five continents, typically found in *K. pneumoniae* ST307 [5]. Plasmid similarity may indicate pKPN3-307_typeA-like plasmids such as pESBL-PH confer limited fitness cost to the host bacterium. Indeed, pKPN3-307_typeA-like plasmids have been detected clonally expanding in different host *K. pneumoniae* STs. A pKPN3-307_typeA-like plasmid, Plasmid A, was responsible for half of all ESBL infections during a 1-year plasmid surveillance study across hospitals in Australia [7]. The homology between Plasmid A and pESBL-PH-56 may indicate that pESBL-PH-56 can expand its host range and persist in the hospital environment.

The *K. pneumoniae* ST628 strains harboured pESBL-PH plasmids with a ~15.3-kb IS*26* PCT. The PCT encoded the ESBL gene, *bla*_CTX-M-15_, alongside four other AMR genes. Importantly, both IS*26* copies bounding the resistance genes are in the same orientation, which is essential for the movement of IS26 PCTs [29,30].

High-resolution typing assigned the ST628 strains the hypervirulent (HV) capsule type K2. The K2 capsule has been associated with a HV phenotype that prevents phagocytosis by neutrophils [31]. Indeed, independent typing of outbreak strains by Public Health England (PHE) confirmed a K2 capsule. In a recent recombinant cloning assay, the replacement of the K2 glycosyltransferase *wcaJ* gene from the capsular polysaccharide (CPS) operon into the K1 knockout *wcaJ K. pneumoniae* strain K2044 was associated with uronic acid production levels similar to the parental strain K2044(K1), but not those of the K1 strain replaced with the K64 *wcaJ* gene, thereby confirming that the K2 CPS gene *wcaJ* could form a hypercapsule [31]. Uronic acid is the major component of the capsule. The K2 *wcaJ* gene replacement strain also displayed mucoviscosity levels similar to the K2044^K1wcaJ^ but not those observed with the K2044^K64wcaJ^ strain. The recombinant K2044 *wcaJ* K2 strain, K2044^K2wcaJ^, exhibited serum resistance levels similar to K2044^K1wcaJ^ but significantly higher than K2044^K64wcaJ^ strains [32]. The K2 capsule may contribute towards the virulent potential of the ST628 strains, potentially assisting their survival and ongoing dissemination.

Relative to other virulent K2 strains, the sequenced ST628 strains harbour a few virulent factors. Previously reported K2 strains, which demonstrate enhanced lethality in mice, additionally encode key virulence factors beyond *iutA* encoded by the ST628 strains, including *iroN* and *rmpA* found in K2 ST65, -86, and 373 *K. pneumoniae* strains, respectively [33]. Despite this, the polysaccharide capsule remains a key virulence factor in *K. pneumoniae* strains. Capsule switch strains have recently demonstrated the K2 capsule promotes *K. pneumoniae* survival in the bloodstream. The capsule provides protection against liver resident macrophage Kupffer cells (KCs) [34]. The K2 capsule may, therefore, represent a key virulence factor in the ST628 strains and may contribute to enhanced dissemination. However, the ST628 strains may be less virulent than another uncommon *K. pneumoniae* CTX-M-15 ST1788 strain that caused a nosocomial outbreak in the UK. The ST1788 strain additionally encoded the yersiniabactin locus, a key virulence factor responsible for scavenging iron from host transport proteins, which facilitates *K. pneumoniae* survival in the host [12].

The outbreak strain does however possess multiple genes linked to environmental persistence, including thermotolerance, heavy metal and ultraviolet resistance. Notably, the outbreak plasmid encoded a heat shock protein ClpK. Mutagenesis and plasmid analyses reveal plasmid-encoded *clpK* increases the survival of *K. pneumoniae* strains at elevated temperatures of 58/60 °C [35]. ClpK produced by outbreak *K. pneumoniae* isolates may enable the survival of bacteria during common disinfection protocols, promoting persistence in the hospital environment.

A single clonally related K2 ST628 *K. pneumoniae* strain with a related MDR pESBL-PH plasmid (pESBL-PH-4) was discovered in 2022, 4 years after the major outbreak in 2018. The colonisation of the hospital environment may have led to the emergence of the ST628 strain. Hospital surfaces can frequently harbour MDR strains, including those which encode *bla*_CTX-M-15_. These have been associated with clonally-related strains causing patient infections [36].

The ST628 strain was obtained from 83.3% of ESBL *Klebsiella pneumoniae* screening samples from patients, indicating the strain may have a high colonisation rate. Indeed, the estimated prevalence of colonisation with CTX-M ESBL-producing Enterobacteriaceae (ESBLPE) in the community in England has been reported at 7.3% and up to 16.% in Birmingham [37]. Importantly, three further cases were identified in the Emergency department (ED) (Figure 1). This may suggest the strain is present in the community and may re-emerge upon admission of patients, thereby emphasising the value of future surveillance of ST628 and the associated pESBL-PH plasmid. A CTX-M-15 encoding 180-kb IncFIIK plasmid in *K. pneumoniae* ST17 has been stably maintained for up to 2 years in colonised patients [38]. Furthermore, a recent study has demonstrated the long-term transmission of AMR, including ESBL genes, between healthcare and community networks can be facilitated by the transmission of plasmids circulating across niches [39]. High colonisation rates, combined with long-term persistence and plasmid transfer, may engender a scenario for future dissemination of ST628 and its CTX-M-15 encoding plasmid. Active surveillance should also be motivated by the discovery of phylogenetically close strains harbouring almost identical plasmids in distinct geographical areas and temporal periods, including Spain and the USA.

Despite the presence of a K2 capsule, additional analyses measuring uronic acid levels, hypermucoviscosity, and *Galleria mellonella* larvae infection assays are required to precisely determine the virulence status of the outbreak strain relative to *K. pneumoniae* K1/K2 and non-K1/K2 control strains. Such analyses will place the virulence of the strains into context, as has been reported with other *K. pneumoniae* strains with a K1/2 capsule [34]. Furthermore, only 10 strains were sequenced using nanopore and illumina platforms. Deeper sequencing may have enabled the determination of potential transmission routes. Despite this, we performed high-resolution sequencing of the isolate with the earliest collection date, alongside isolates collected from unique wards in 2018 and an additional isolate detected in 2022. The narrow SNP distance between all of these strains confirmed a single clone has spread across multiple hospital departments. In addition, nanopore-only assemblies yielded strains with the same ST and plasmids of almost identical size, suggesting a single strain was responsible for the outbreak.

## 6. Conclusions

MDR ST628 *K. pneumoniae* strains were identified from a nosocomial outbreak at a UK district general hospital. Evidence of spread between hospital wards was discovered. The rare ESBL ST628 strains encoded an ~247-kb FIB(K) plasmid encoding 11 AMR genes: *aac(3)-IIe*, *aac(6*′*)-Ib-cr*, *strA*, *strB*, *bla*_CTX-M-15_, *bla*_OXA-1_, *bla*_TEM-1B_, *dfrA14*, *qnrB1*, *sul2*, and *tet(A)*. This plasmid closely resembles plasmids from diverse international regions, including phylogenetically related strains, which date back to 2012. Future surveillance of *K. pneumoniae* ST628 and the associated pESBL-PH plasmid may be necessary to detect and manage a future outbreak. This work also provides an improved understanding of CTX-M-15-producing *K. pneumoniae* strains of epidemic potential in the UK.

## Figures and Tables

**Table 1:** Common Antimicrobial Resistance, Virulence and Persistence genes typed in the Nanopore illumina ST628 *K. pneumoniae* Chromosomes.

Category	Functional Group	Gene(s)	Function (Derived from UniProt)
Antimicrobial resistance	Antimicrobial resistance genes	*blaSHV-148*	Penicillin resistance
	Multidrug transporters	*macAB*	Part of the tripartite efflux system MacAB-TolC
		*oqxAB*	Efflux pump membrane transporter
		*MexAB-OprM*	Multidrug resistance efflux pump
		*acrAB*, *acrEF*, *tolC* *acrAB* transcriptional activators: *(marA*, *soxS*, *rob)*	Multidrug transporters/Outer membrane channel. The genes are involved in the export of antibiotics and other toxic compounds from the cells, and help confer a resistance phenotype towards beta-lactams, aminoglycosides, tetracyclines, fluroquinolones, chloramphenicol, acriflavine
		*mdtABCD*, *mdtG*, *mdtH*, *mdtJI*, *mdtK*, *mdtL*, *mdtM*, *mdtNO*	Efflux pumps, implicated in antibiotic resistance. Confers resistance to drugs including fluoroquinolones
Virulence Factors	Fimbria adhesins	*fimBEAICDFGHK*	Type 1 fimbria. Mediate biofilm formation, adhesion to host cells and other bacteria
	Fimbria adhesin	*mrkD*	Type 3 fimbrial adhesion protein
	Biofilm formation	*ecpRABCDE*	*E. coli* common pilus, contributes to the colonization and infection of human tissues
	Siderophores	*entABCDEFHS*	Enterobactin, iron sequestration
		*fepABCDGS*	Ferric Enterobactin transport
	Siderophore receptor	*iutA*	Aerobactin receptor, iron sequestration receptor
	Virulence factors	*pgaABC* *treC*, *BamB (Yfgl)*	Capsule synthesis and biofilm formation, physical attachment of bacteria to surfaces.
Persistence Factors	Heavy metal resistance and homeostasis	*chrB*	Chromate resistance and transport
		*cpxRA*, *cusF*, *cutC*, *cueO*, *cueR*, *copA*	Copper binding response/homeostasis/resistance. Enables binding to hydrophobic surfaces during biofilm formation (CpxA).
		*cusRS*	Copper/Silver regulation/tolerance
		*zraPSR*	Zinc/Copper resistance/tolerance
		*zntA*	Confers resistance to zinc, cadmium and lead.
	Metalloid resistance	*arsBC*, *yffB*	Arsenate reductase, arsenic efflux pump
	Ultraviolet protection	*umuCD*	UV protection, error prone DNA polymerase involved in translesion repair

**Table 2:** Antimicrobial Resistance, Virulence and Persistence genes typed in the Nanopore illumina pESBL-PH plasmids.

Category	Functional Group	Gene(s)	Function (Derived from UniProt)
Antimicrobial resistance	Antimicrobial resistance genes	*aac(3)-IIe*	Aminoglycoside resistance
		*aac(6′)-Ib-cr*	Aminoglycoside resistance
		*strA*	Aminoglycoside resistance
		*strB*	Aminoglycoside resistance
		*bla*_CTX-M-15_	ESBL, resistance to cephalosporins
		*bla*_OXA-1_	Beta-lactamase
		*bla*_TEM-1B_	Broad-spectrum beta-lactamase. Confers resistance to penicillins and first generation cephalosphorins
		*dfrA14*	Dihydrofolate reductase confers trimethoprim (TMP)-resistance
		*qnrB1*	Quinolone resistance
		*sul2*	Sulfonamide resistant dihydropteroate synthase. Sulfonamide resistance.
		*tet(A)*	Tetracycline efflux pump
Virulence Factors	Iron acquisition	*fecIRABCDE*	Ferric citrate transport system
	Outer membrane protein	*traT*	Complement resistance
Persistence	Heavy metal resistance/homeostasis	*cusCFBA*	Efflux system. Mediates copper resistance
		*cusRS*, *pcoE*, *silP*	Copper tolerance/sensing
		*pcoC*	Copper resistance
		*copA*	Copper-exporting P-type ATPase. Exports Cu^+^ from the cytoplasm to the periplasm.
		*arsBC*, *arsH*	Arsenate resistance and cellular excretion
	Thermotolerance	*clpK*	Nosocomial Persistence, resistance to o lethal heat shock
	UV	*umuCD*	UV protection, error prone DNA polymerase involved in translesion repair

## Data Availability

Data are contained within the article and Supplementary Materials.

## Supplementary Materials

The following supporting information can be downloaded at: https://www.mdpi.com/article/10.3390/microorganisms12050883/s1. Supplementary Figure S1: 15.3-kb pseudo-compound transposon (PCT) bracketed by IS*26* insertion sequences.
