# Supplementary figures and images for: Genetic Analyses of Rare ESBL ST628 Klebsiella pneumoniae Detected during a Protracted Nosocomial Outbreak in the United Kingdom
Source: Microorganisms. 2024 Apr 28;12(5):883. doi: 10.3390/microorganisms12050883 (PMC11124425; doi:10.3390/microorganisms12050883)

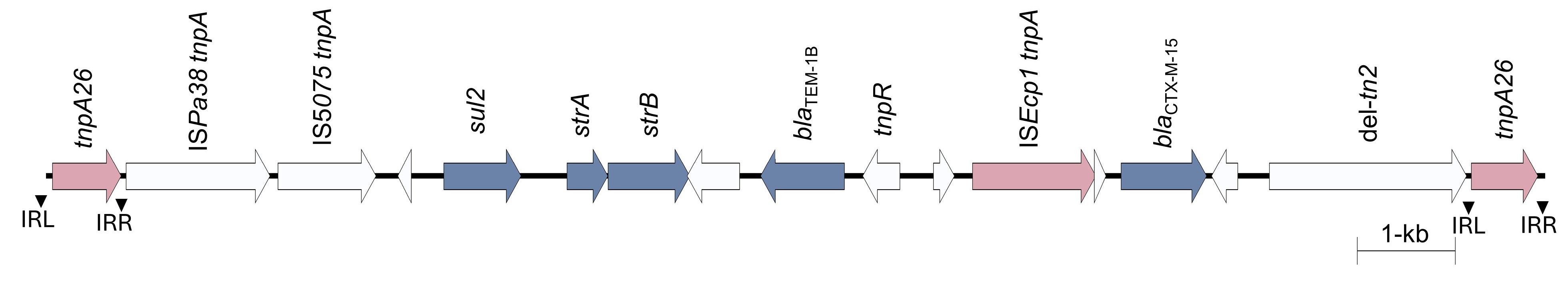

Supplement: Supplementary file 1 [file microorganisms-12-00883-s001.zip › Supplementary Figure S1.jpg]
